# A New Method of Applying Pressure in Traumatic Aneurism

**Published:** 1880-12

**Authors:** 


### A NEW METHOD OF APPLYING PRESSURE IN TRAUMATIC ANEURISM.

Dr. B. R. Palmer, of Sauk Centre, Minn., reports a case of traumatic aneurism of the femoral, resulting from a knife-wound, in which he made an ingenious use of the plaster-of-Paris bandage to facilitate the application of pressure to the artery. He took a band of coarse, thick Mackinaw flannel about six inches wide and long enough to envelop the thigh and lap over about two inches, soaked it in a mixture of the plaster, and applied it to the thigh over the tumor. An aperture was cut in this band, directly opposite that part of the femoral artery just below the profunda, where the pressure was desired. As soon as the plaster had set, about twenty minutes, a piece of cork, properly shaped and covered with chamois-skin, was pressed down upon the artery through this aperture, about an inch of the cork being allowed to project outside the band. A roller-bandage of stout elastic webbing was then applied around the thigh, outside the plaster band, and over the projecting portion of the cork compress, the tension being increased at every turn, until pulsation in the popliteal space could be no longer felt. The apparatus was allowed to remain in situ for about twenty-four hours, with very little inconvenience to the patient. On loosening it there was no return of pulsation and coagulation of the contents of the tumor seemed to have taken place. In a few weeks the tumor had become absorbed and gave the patient no trouble. Dr. Palmer has also employed this apparatus with great success in the treatment of secondary hemorrhage from gunshot wounds and after amputations, particularly those from frost-bites, where secondary hemorrhage so frequently occurs, and where long-continued pressure is necessary.—*Chicago Medical Review*, Sept. 5.

